# Thioetherification of Br-Mercaptobiphenyl Molecules on Au(111)

**DOI:** 10.1021/acs.nanolett.2c04619

**Published:** 2023-02-10

**Authors:** Ana Barragán, Roberto Robles, Nicolás Lorente, Lucia Vitali

**Affiliations:** † Donostia International Physics Center (DIPC), Paseo M Lardizabal 4, 20018 San Sebastián, Spain; ‡ Advanced Polymers and Materials: Physics, Chemistry and Technology, Chemistry Faculty (UPV/EHU), Paseo M Lardizabal 3, 20018 San Sebastián, Spain; § Centro de Física de Materiales CFM/MPC(CSIC-UPV/EHU), Paseo M Lardizabal 5, 20018 San Sebastián, Spain; ∥ Ikerbasque Research Foundation for Science, Plaza Euskadi, 5, Bilbao 48009, Spain

**Keywords:** Thioether synthesis, On-surface chemical reaction, Scanning tunneling microscopy and spectroscopy, Density functional theory

## Abstract

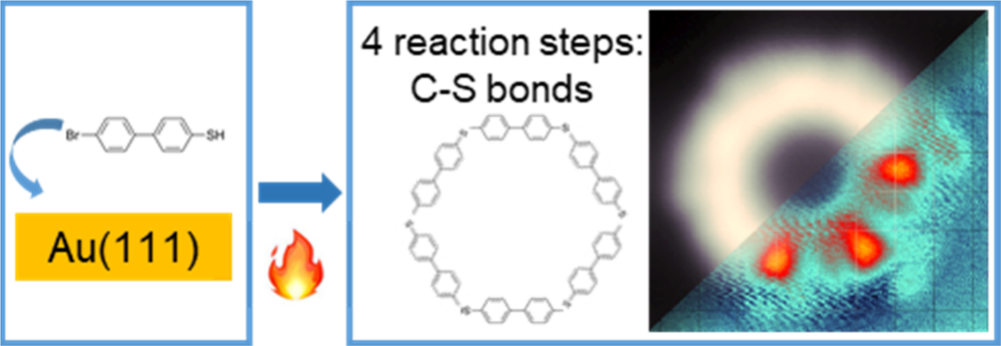

Thioether polymers are fundamental for a variety of applications. Their synthesis is, however, more challenging than that of other metal-catalyzed reactions due to the reported detachment of the S atom during thermal activation. In this study, it has been demonstrated unambiguously that thermal annealing results in the thioetherification of the 4-bromo-4-mercaptobiphenyl molecule (Br-MBP) adsorbed on the surface of Au(111). Through complementary techniques, such as scanning tunneling microscopy, spectroscopy, and first-principle calculations, we have identified four reaction steps, involving sulfhydryl or bromine molecular functional groups and leading to the formation of intermolecular C–S bonds. To form the thioether polymer and to overcome the competitive formation of C–C bonds, two reaction steps, the dehalogenation, and dissociation of the S–Au bond, must occur simultaneously. We detail the electronic properties of the phenyl–sulfur bond and the polymer as a function of the ligand length. This result suggests a wider perspective of this chemical synthesis.

Sulfur-containing polycyclic aromatic hydrocarbons are fundamental for a variety of applications such as nanoelectronics, optoelectronics, catalysis, biology, or pharmaceutics.^1^ The formation of thioether structures increases the mechanical strength, improves energy storage and charge conductance, favors light absorption, enables tunable electroluminescence emitters, and favors (bio)-catalytic activities, such as those observed in enzymes or proteins and macrocycles.^2−9^ The metal-catalyzed cycloaddition of S-rich molecules faces, however, greater difficulties than other reactions. The strength and coordination of this chalcogen atom with traditional metal catalysts, such as Au, Ni, Pd,^1,2,10−12^ easily induce its detachment from the organic structure eventually poisoning the catalyst surface. Therefore, the on-surface synthesis of such polymers is usually limited to the C–C coupling of prefunctionalized molecular aryls containing O, N, or S atoms,^3^ while the direct involvement of chalcogen atoms in intermolecular covalent bonds remains challenging.

Here, we show that the synthesis of thioether polymers by −C–S– intermolecular coupling can be successfully achieved on the surface of Au(111), despite the affinity of sulfur with gold. Using scanning probe techniques at low temperatures and density functional theory (DFT) calculations, we describe the formation of thioether bonds by characterizing the thermal activation of functional groups of 4′-bromo-4-mercaptobiphenyl (Br-MBP) molecules adsorbed on this metal surface (Figure 1). The double functionalization of the Br-MBP precursors with a Br and a sulfhydryl terminal group is likely fundamental for mastering the described difficulties in the C–S etherification. On its own, each of these two molecular terminations follows two of the most extensively characterized reaction schemes that lead to strong chemical bonding. Their simultaneous presence on the same reaction template, however, unexpectedly modifies these paths. The synthesis of a different and specific product, namely, the intermolecular −C–S– bonding, shows that the S–Au–S bonds, spontaneously formed when Br-MBP molecules are adsorbed on gold surfaces at room temperature,^13^ as well as the thermally activated Ullmann C–C coupling reactions that follow molecular debromination are overcome. Thus, the proximity of both molecular ending groups and likely the simultaneity of some of the four identified reaction steps must be at the origin of this modified reaction mechanism, as will be here discussed.

**Figure 1:**
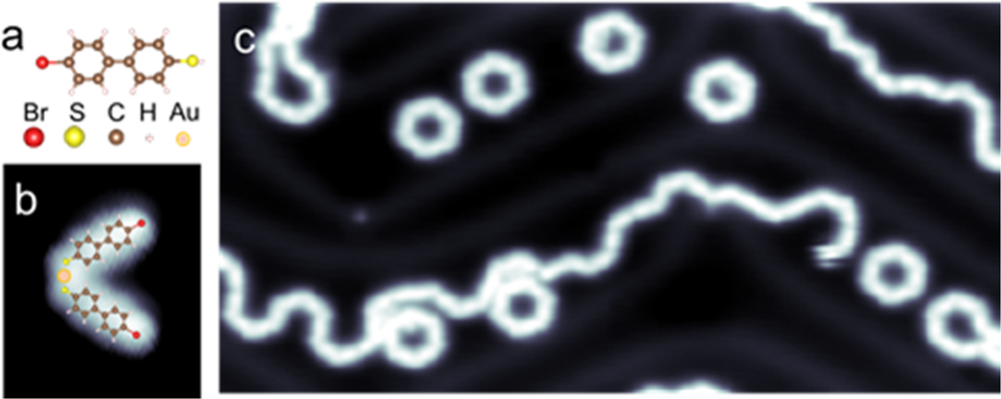
Thioether structures formed on Au(111) surface. a. Sketched molecular structure. b. Topographic image of a Br-MBP dimer (3 × 3.5 nm^2^), c. Topographic image of the polymer structures formed after the annealing of 4′-Bromo-4-mercaptobiphenyl (Br-MBP) molecules (30 × 15 nm^2^; 1 V, 0.3 nA).

In Figure 1, a representative topographic image shows extended zigzagging chains and closed-loop structures formed after the adsorption and annealing of the Br-MBP molecules on Au(111) at 180 °C. Among these, the smallest ring-like structures are hexamers, although heptamers and octamers are not uncommon, suggesting that their formation is not determined by the high-symmetry directions of the substrate.^14^

A careful structural characterization of these assemblies provides already the first hint that these structures cannot be described according to the Ullmann and Au-disulfide reaction schemes mentioned in literature^13,15−19^ and expected for the two individual terminal groups. Indeed, the 1.1 nm long molecular segment included between two subsequent bents in chains and loops is only about the size of the biphenyl group of one pristine MBP molecule. This critical length is too short to justify the formation of the extensively characterized Ullmann C–C cross-coupling reaction, although the thermally induced detachment of the halogen atom is observed (Supporting Information SI2).^15−19^

In the absence of this new C–C bond, the formation of the observed chains and loops can only be explained by considering the direct involvement in the assembly of the other molecular termination, i.e., the sulfur side. However, such a head-to-tail assembly of all molecules upon the annealing requires a considerable structural and chemical change in the assembly. Indeed, when adsorbed on Au(111) surface, the Br-MBP molecules spontaneously dehydrogenate the sulfhydryl group and form dimer pairs bridged by strong S–Au–S interaction already at room temperature^11,13,20−22^ (Figure 1b).

Evidence of a change in the S–Au bonding interaction between the molecules and the substrate is provided by their displacement with the tip of the scanning tunneling microscope in dimers and in polymers (Supporting Information SI2). Indeed, before the annealing, this strong anchoring point enabled, at most, to change the assembly of the Br-MBP dimer from a cis to a trans configuration by rotating one of the molecules around the S bonding.^13^ The full displacement of the annealed MBP structures, as shown in the Supporting Information SI2, confirms the weakening of the S–Au interaction. Furthermore, and perhaps most importantly, the integer manipulation, i.e., without causing the constituent elements to separate, corroborates the polymerization of the Br-MBP molecules.

In Figure 2, we compare the electronic properties of the annealed Br-MBP/Au system with those calculated by Density Functional Theory (DFT) for a model structure that considers our hypothesis on the assembly configuration of the polymer. In panels b and c, the density of states is displayed as color scale representations of a series of d*I*/d*V* spectra measured along the blue and yellow lines drawn on the topographic image. The variation of the electronic properties, highlighted by the dotted lines, reflects the distinct character of the molecular segments and bending positions observable also in the correspondingly d*I*/d*V* maps (f–h panels). The remarkable agreement between experimentally measured and calculated density of states allows us to associate the major contribution to the density of states at the lowest measured energy (panels f and j) to the S and phenyl region. A clear distinct fingerprint of the −C–S–C intermolecular bonds is observed instead at −1.5 V (panels g and k). The intriguing helical structure seen in panels h and l provides evidence that the molecular adsorption configuration in the thioether structure is not perfectly planar (Supporting Information S1 and S3). This electronic structure is caused by the electrostatic repulsion and steric hindrance between hydrogen atoms at the edges of subsequent phenyl arenes. Overall, it is worth noting the spatial periodicity of the density of states along the polymer. Each molecular segment exhibits the same electronic structure, which validates the proposed polymer assembly (Figure 2i and Supporting Information S1 and S3).

**Figure 2:**
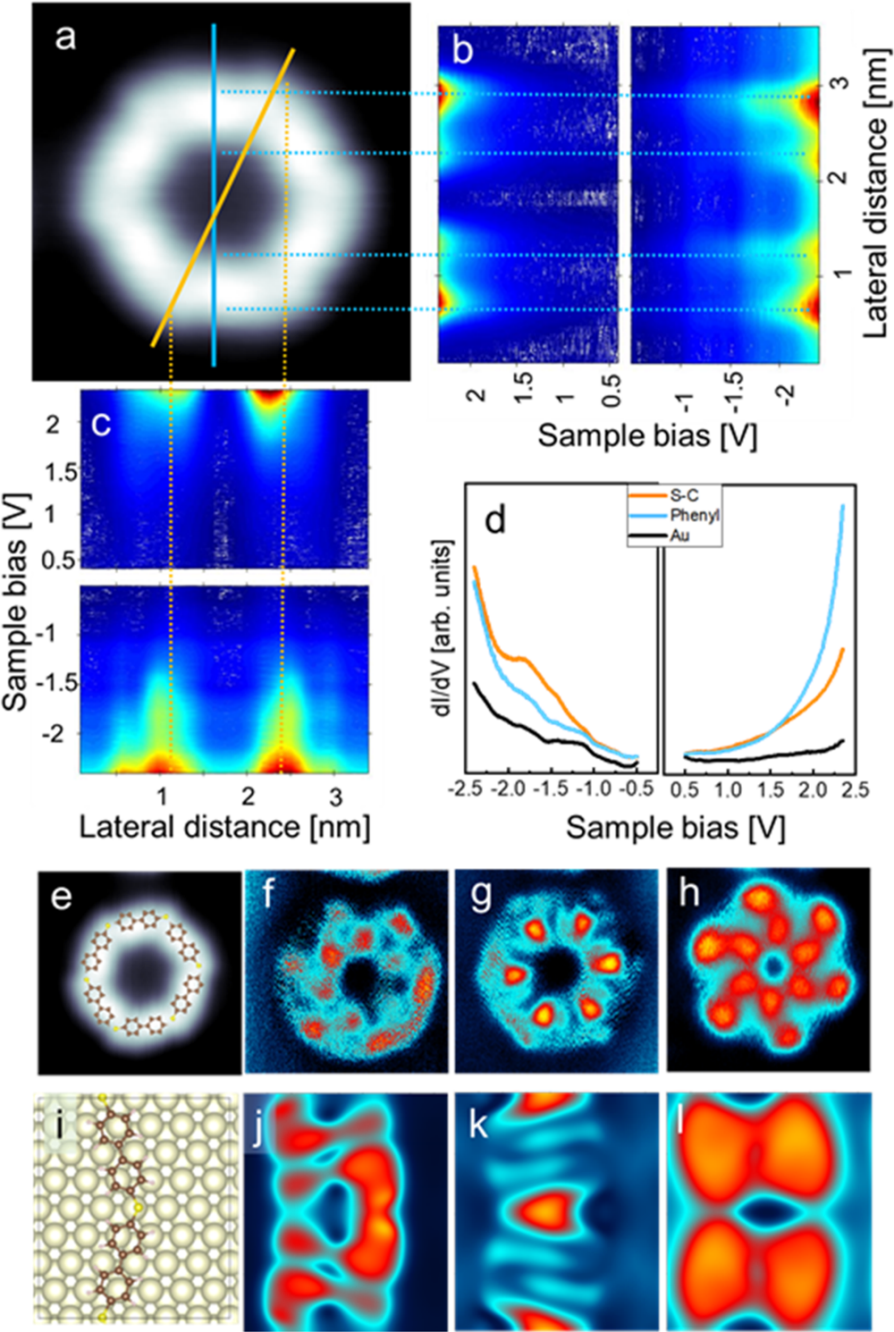
Electronic properties of the polymeric structure. a. Topographic image (size 4 × 4 nm; −0.4 V, 0.5 nA). Color scale representations of d*I*/d*V* spectra measured along the b. blue and c. yellow lines in panel a, respectively. d. d*I*/d*V* spectra at selected positions. e. Topographic and d*I*/d*V* maps at f. −2.1 V; g. −1.5 V; h. 2.1 V and their comparison with the theoretical simulated distribution of states in the modeled structure The simulations are not done for a cyclic polymer, but the local structure is largely reproduced. i. j. −1.85 V; k. −0.97 V; l. 1.83 V.

Our experimental and theoretical data have identified four steps of this polymerization reaction that will be discussed here. The adsorption and interaction of Br-MBP molecules with an Au(111) surface at room temperature induce the first reaction step, i.e., *i*. *The dehydrogenation of the sulfhydryl termination and the formation of Au-thiolated molecular pairs,* as previously demonstrated.^13^ This is consistent with similar sulfhydryl molecular terminations in mercapto or thiophenyl molecules. All adsorbed molecules are involved in this reaction, which is followed by the formation of molecular dimers bonded through the S–Au–S interaction. This reaction is already taking place at room temperature.^11,13,20−22^ Thermal activation is required instead for the following three reaction processes: i.e. *ii*. *The molecular debromination process; iii*. The *reductive scission of the Au–thiol bond* with the detachment of the Au adatom and the separation of the two MBP molecules, which must precede the *iv. Carbon–sulfur coupling and the formation of a thioether polymer*.

Considering at first the effect of temperature on these molecular terminations, dehalogenation is, perhaps, the most successful surface-catalyzed reaction. In the Ullmann oxidative-addition and reductive-elimination scheme,^15^ the detachment of the halogen atom commonly leads to covalent C–C coupling between molecules. This reaction is indeed commonly used for the synthesis of graphene nanostructures on surfaces.^3,17,18^ As a result, the overcome of this reaction scheme and the observation of the C–S bond, shown by the scanning tunneling topographic image of Figure 1 (see Supporting Information SI2), is a novel finding.

Similarly, annealing of a variety of mercapto or thiophenyl molecules forming Au-thiolated bonds at temperatures of about 230–250 °C, thus even higher than the one used in the present work (180–200 °C), has not modified the gold disulfide bridge.^11,22^ As the temperature increases further, these structures undergo the cleavage of the chalcogen-carbon bond.^11,22^ Consequently, the S atom separates from the molecule but it remains bound to the Au atom. Eventually, the C–C coupling between desulfurated molecular units can be observed.^11,12,23^ Clearly, in the present case, the atomic scission of S does not take place.

Some additional insights into the origin and selectivity of this unexpected reaction can be gained considering the simultaneous presence in the reaction template of the reacting halogen termination and S–Au bond. Notwithstanding the considerably different binding energy of the C–Br (76 kcal/mol)^24^ and Au–sulfur bonds (100 kcal/mol),^24^ the proximity of the reactants could explain the head-to-tail assembly. Additionally, to outweigh the competing formation of C–C bonds, the C–Br and S–Au bonds must break simultaneously to favor molecular thioetherification. In fact, no other intermediate chemical configurations, such as either isolated molecules or isolated molecules resulting from the dissociation of the Au-disulfide bridge or dehalogenated dimers have been observed.

In this context, it is worth recalling that C–S coupling can be obtained in solution catalyzing the oxidative addition and reductive elimination of an aryl halide to sulfhydryl molecules with Fe, Co, or Ni transition metal atoms to form a thioether.^10,23,25^ In our reaction, the affinity of sulfur for gold causes at first the formation of a strong S–Au bond. This bond and its dissociation constitute two remarkable and important steps observed in the present thioetherification reaction that are not described by previous reaction schemes.^10,23,25^

In spite of the very large reproducibility of the described polymeric structure, a C–C cross-coupling reaction can punctually occur when one S atom is missing. A straight segment of the closed loop polymer with double length is indicated by the arrow in Figure 3a. The structure’s submolecular resolution shows that this segment is constituted by 4 phenyls, followed by sulfur atoms, which are seen as black dots in Figure 3 b. The density of states along the 4-phenyl segment differs from the others. An electronic structure at about 2.2 V, is clearly seen both in the d*I*/d*V* spectrum and in the d*I*/d*V* map (Figure 3c–d). A theoretically calculated structure shown in Figures 3e–f supports such a modification of the density of states. Thus, the electrical conductance of the polymer can be tuned by an appropriate choice of the molecular precursor length, while maintaining the etherification scheme.

**Figure 3:**
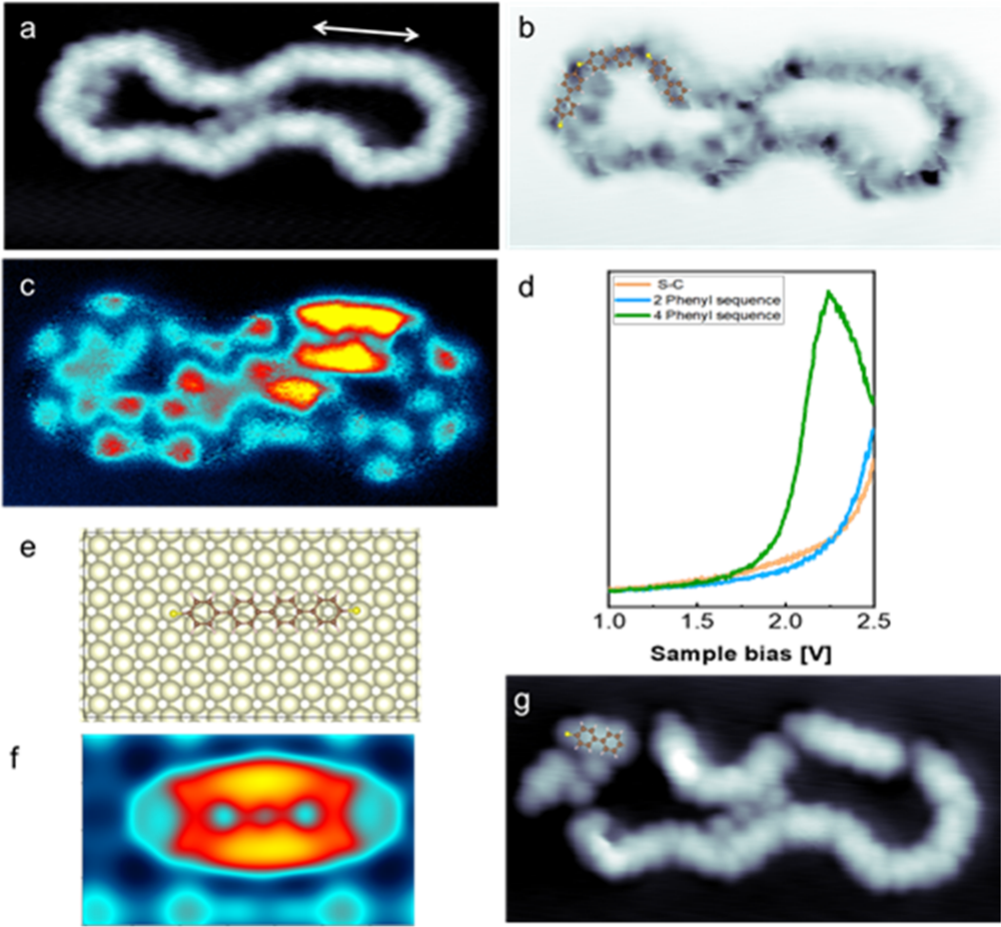
Tetraphenyl thiol structure and electronic properties. a. Topographic image of a large closed loop structure enclosing few Br atoms (−0.4 V, 1 nA). The arrow indicates a long straight segment of the loop b. High-resolution topographic image with superposed structural model. A sequence of 4 phenyls can be recognized in the long straight segment. c–d. d*I*/d*V* map at 2.2 eV and spectra at selected positions. e–f. DFT simulation of a tetraphenyl molecule with S terminations on Au(111): model and d*I*/d*V* map (1.83 V). g. Topographic image of the structure after the rupture of several C–S bonds. Single debrominated monomers and a 4 phenyls-thiol can be identified (Images: 8 nm × 4 nm).

The rupture of the C–S bonds can be induced by scanning the surface at 2.7 V. This voltage separates the polymer into its components, as shown in Figure 3g. Here, the head-to-tail assembly is even clearer when considering the distinctive shape of each unit, where the S atom and the bi- or tetraphenyl structures are identified by one shallow maximum followed by two or four large maxima, respectively. Interestingly, the aforementioned electronic fingerprint of the S bond bridging two phenyls at −1.5 V is exhibited only in the thioether structure but not in the middle of the 4 phenyls segment and in the separated MBP units (Supporting Information SI5).

In conclusion, we have described the synthesis of a thioether polymer on Au(111) surface by chemical reaction of Br-mercaptobiphenyl molecules. Our combined experimental and theoretical investigation has allowed identifying four reaction steps through which both terminal groups of the pristine molecules must undergo to polymerize. We speculatively hypothesized that the close proximity of these molecular groups promotes the observed and exclusive intermolecular bonding. Indeed, two of the reaction steps, i.e., the dehalogenation of the phenyl-bromine and the dehydrogenation of the sulfhydryl, which drive to well-known reaction products in molecules containing either of these functional groups, are here only intermediate steps of the reaction. Clearly, the Br-MBP molecules′ thioetherification requires the dissociation of the strong Au-sulfate bond, which is not achieved by thermal annealing alone. Similarly, the extensively characterized C–C coupling that is frequently reported upon the annealing of Br-functional groups is outweighed by the exclusive synthesis of C–S bonds. Our findings provide new insight into the synthesis of these structures, whose electronic properties can be tuned based on their ligand’s length.

## Supporting Information Available

The used experimental and theoretical methods, additional information regarding the structural changes upon thermal annealing of the molecules adsorbed on the Au(111) surface, their manipulation, the energetics of closed-loop structures, and the electronic properties of heptameric molecular loops, and the electronic properties of C–S bonds (PDF)
