# Supplementary material for: Thioetherification of Br-Mercaptobiphenyl Molecules on Au(111)
Source: Nano Lett. 2023 Feb 10;23(4):1350–4. doi: 10.1021/acs.nanolett.2c04619 (PMC9951239; doi:10.1021/acs.nanolett.2c04619)
Supplement: Supplementary file 1 — nl2c04619_si_001.pdf [file nl2c04619_si_001.pdf]

Supporting information

Thioetherification of Br-mercaptobiphenyl molecules on Au(111)

Ana Barragán1,2,3, Roberto Robles 3, Nicolás Lorente1,3, Lucia Vitali1,2,3,4

1. Donostia International Physics Center (DIPC), Paseo M Lardizabal 4, 20018 San Sebastian
2. Advanced Polymers and Materials: Physics, Chemistry and Technology, Chemistry Faculty (UPV/EHU), Paseo M Lardizabal 3, 20018 San Sebastian
3. Centro de Física de Materiales CFM/MPC(CSIC-UPV/EHU), Paseo M Lardizabal 5, 20018 San Sebastián
4. Ikerbasque Research Foundation for Science, Plaza Euskadi, 5, Bilbao 48009

1. **Experimental and theoretical methods**
2. **Adsorption of Br-mercaptobiphenyl molecules on Au(111), formation and manipulation of thioetherified polymers**
3. **Energetics of closed loop structure**
4. **Electronic properties of an octamer closed-loop and chain structures**
5. **Ph-S-Ph: electronic property**
6. **References**

1. Experimental and theoretical methods

1a. Sample preparation and experimental characterization.

The 4´-Bromo-4-mercaptobiphenyl (Br-MBP) molecules have been deposited in ultra-high vacuum conditions (UHV) onto the Au(111) surface previously prepared by cycles of Ar+ ion sputtering and subsequent annealing. A post-annealing of the molecules adsorbed on the Au surface at a temperature in the range of 180-200ºC leads to the formation of C-S covalent bonds between phenyl arenes and to the molecular thioetherification. After the annealing, the sample was transferred in UHV into a bath cryostat at the temperature of 1K to perform scanning tunneling microscopy and spectroscopy measurements.

1b. Theoretical simulation. Electronic structure calculations were performed in the framework of the density functional theory (DFT) as implemented in VASP [1,2]. We used the projector augmented-wave method [3] to treat the core electrons and we expanded the wavefunctions by means of a plane-wave basis set with an energy cutoff of 400 eV. We used the PBE functional [4] to treat the exchange-correlation energy. We added the missing van der Waals interactions using the Tkatchenko-Scheffler scheme [5]. We modeled the Au (111) surface using a four-layer slab. The simulation of the systems shown in this work presents big challenges due to the size of the unit cells needed to simulate the molecular closed loop structures and to the experimental poor orientation periodicity of the molecules in the chains. In order to capture the main effects we have used the periodic zigzag chain shown in Figure SI1. The advantage of this geometry is that it allows us to use a relatively compact rectangular unit cell (6x4) to simulate a chain of molecules forming an angle of about 144º, which would correspond to the angle in a 10-unit "ring". The position of the S atoms was chosen to be on top of Au atoms, as seen experimentally (see following paragraph 4). With this configuration we expect to capture the main features of both closed loops and chains adsorbed on the Au(111) surface. All atoms except the lower two Au layers were relaxed until all forces were smaller than 0.02 eV/Å. We have simulated the STM images within the Tersoff-Hamann approximation [6] using the method by Bocquet et al. [7] as implemented in STMpw [8]. In most calculations, we have used a (3x3x1) k-grid sampling. A finer (5x5x1) grid was used to plot the dI/dV maps. Images of DFT ball and stick models were generated using the VESTA code [9].

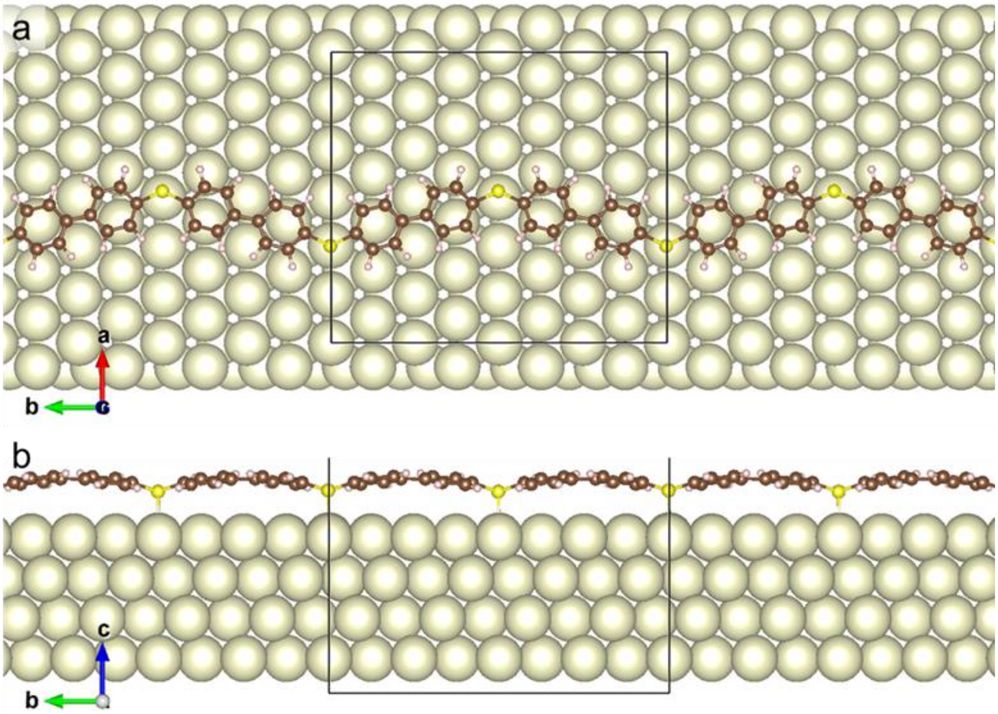

Figure SI1. Relaxed geometry of a zigzag chain. a. Top and **b.** side views of the structure. The unit cell used in the calculations is shown with black lines.

In this adsorption configuration, the phenyl arenes in the chains conserve some degree of twist with respect to the surface plane. This twist causes an uneven surface interaction of the C-H atoms at either side of each phenyl structure. This effect becomes evident in the electronic properties and justify the observed chiral electronic structure in the density of states observed in figure 2h of the main manuscript.

2. Adsorption of Br-mercaptobiphenyl molecules on Au(111), formation and manipulation of thioetherified polymers

In Figure SI2, the adsorption configuration and assembly of the Br-MBP molecules on Au(111) are shown as a function of increasing coverage and temperature. In panels a and b, the molecular coverage is progressively increased, though it remains still in submonolayer regimes. The trend of the molecules to assemble in dimer structures is evident, confirming the reported de-hydrogenation of the sulfhydryl termination of the molecules and the formation of Au-sulfate bonds even at very low coverages [10]. As the coverage increases the isolated dimers assemble in more compact structures (panel b), preferably in the FCC region of the Au(111) reconstruction. The post-deposition annealing causes a major structural change with the induced polymerization of the molecules described in the main manuscript. The Br atoms detached from the molecules are still visible between the polymer chains or trapped in the closed loop structures (panel c) in accordance with previous reports [11].
As the annealing temperature increases and the Br atoms trapped between the polymers reduce (figure SI2c), the thioether polymer detaches from the island. During this process, both long-chain and closed-loop structures form, as shown in figure SI3. When the temperature increases, longer chains detach and move on the Au surfaces resulting in

loops with a greater number of monomers (figure SI3 b and c, and table SI1). The formation of these structures varies as well with the molecular coverage. However, if the MBP coverage approximates the monolayer (figure SI3d), the chains bundle, suggesting that their free movement on the surface is needed for the formation of rings.

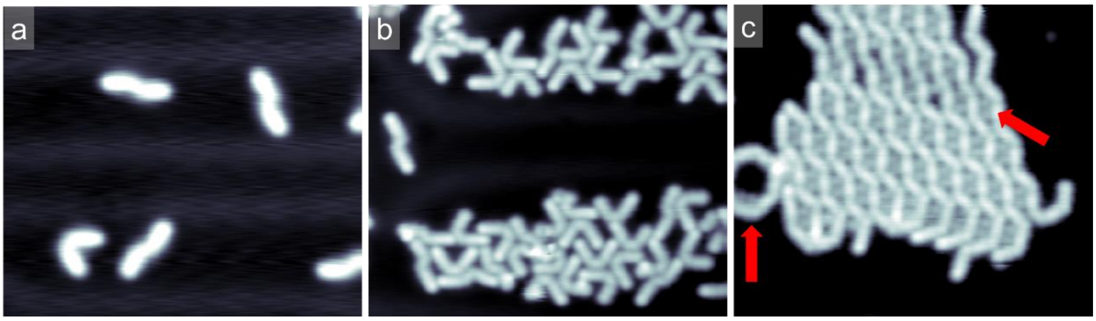

Figure SI2. Structural configurations of Br-MBP molecules on Au(111) surface as a function of increasing coverage **a-b**, and annealing temperature **c**. The red arrows indicate a few of the trapped Br atoms between the polymer chains (size of image 15nmx13nm).

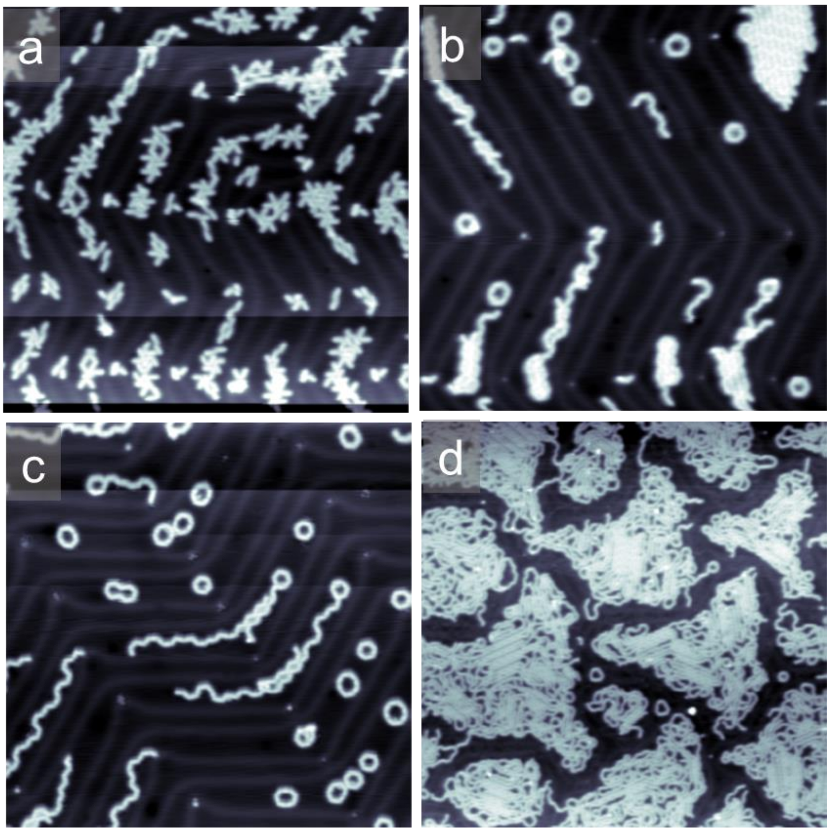

Figure SI3. Formation of closed-loop structures on the surface as a function of increasing temperature at coverages of about 0.25ML a. Room temperature; **b.** 180ºC; **c.** 200ºC; **d.** 0.7 ML annealing at 200ºC. Image size: a.-c. 50nmx50nm, d.100nmx100nm

**Closed loop structures**	**180ºC**	**200ºC**
**hexamers**	75%	54%
**heptamers**	25%	25%
**octamer**		13%
> **8 monomers**		8%

Table SI1: Statistics of closed-loop structures versus annealing temperature at Br-MBP coverages of about 0.25ML. The numbers indicate the size of the closed loops in percentage.

Once formed, the polymers can be manipulated and displaced on the surface by pushing or lifting with the tip of the STM both in the isolated or in the island structure. This manipulation concerns the thioether structure without inducing the separation into its constituent elements (figure SI4) as expected for polymerized molecules bonded through covalent chemical bonds.
The spontaneous separation of the polymers from the islands and their manipulation with the tip of the STM demonstrate that the interaction of the molecules with the supporting substrate is reduced. Indeed, a complete displacement of the molecular dimers before their annealing has not been achieved due to the strong S-Au-S bonds [10].

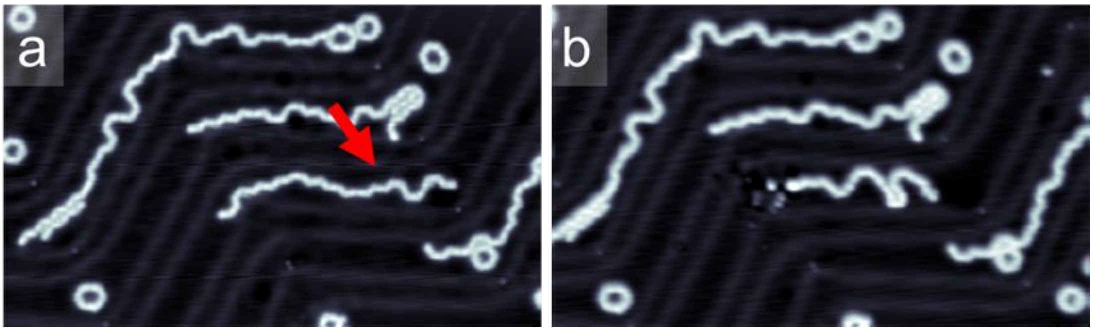

Figure SI4. Manipulation of polymers. a-b. Topographic images of the same surface area (size 50nmx30nm) before and after the manipulation of the polymer are indicated by the arrow.

3. Energetics of closed loop structure

At all measured annealing temperatures, structures formed by a small number of monomers result in closed-loop structures. Among these, hexamers are the most common, although heptamers or octamers and larger loops can be observed. Tetramer and pentamer loops instead have never been observed.

The energetics of such structural configurations has been estimated by density functional theory using gas-phase calculations. In order to be closer to the adsorbed configuration, to build the loop structures we have used the on-surface geometry of the molecule taken from the calculation of the supported zigzag chain (figure SI1). Then, we relaxed the structures keeping fixed the *z* coordinates. The results confirm that the stability of the

system increases with increasing numbers of monomers (Figure SI5). Closed loops including a number of monomers larger than 6 have comparable energy. A possible reason for the higher stability of the larger structures can be found in the steric hindrance of H atoms. The molecules are not perfectly planar in these structures. The degree of twisting of the consecutive phenyl rings induced by the neighborhood of hydrogen atoms increases with the reduced size of the closed loop structure. Thus, this steric effect is likely causing an increased energy cost of formation for the tetrameric and pentameric loops as predicted by our theory calculations.

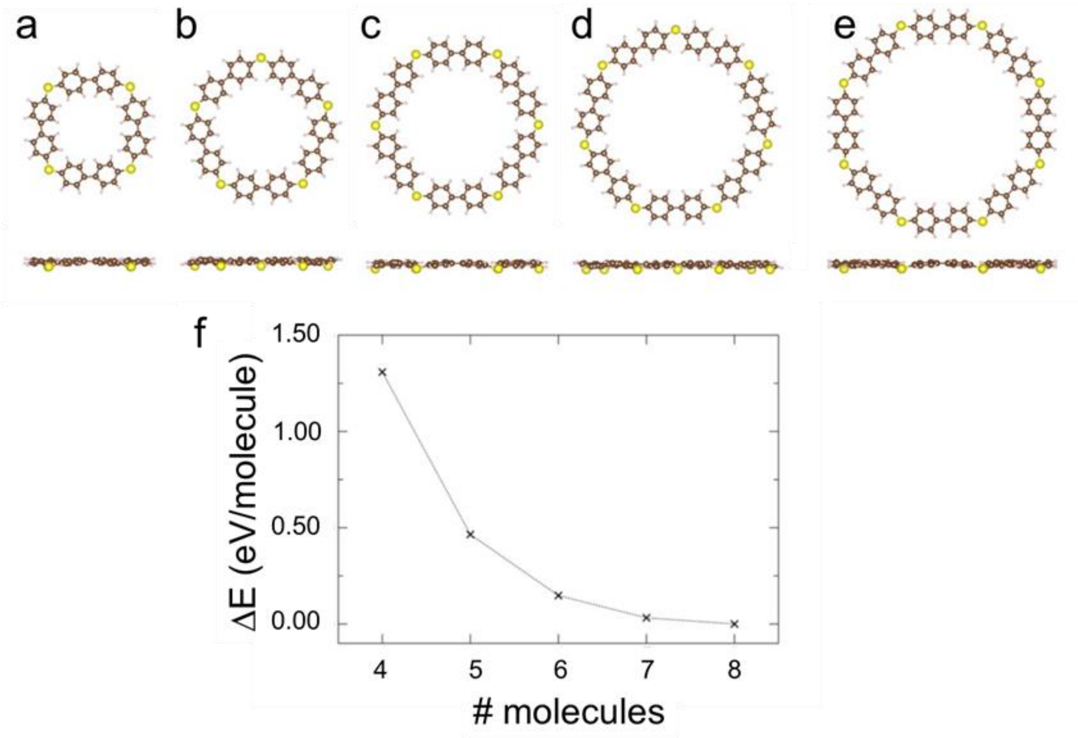

Figure SI5. Density functional calculations of the energy of closed loop structure as a function of an increasing number of MBP members in the gas phase. a-e. top and side view of thioether rings formed by 4 to 8 MBP molecules and **f.** energy of the system.

4. Electronic properties of an octamer closed-loop and chain structures

Independently on the assumed conformation of the polymer, hexamers, larger closed-loop structures or linear elongated shapes have comparable electronic properties. In Figures SI6 and SI7, we show for comparison the energy maps measured on an octamer and chain structure. The characteristic electronic fingerprint of the phenyl and/or of the newly formed S-C, described in the main manuscript, can be recognized in all structures.

In figure SI6d, the atomically resolved image of the Au substrate, superposed to the molecular topographic image (panel a) and to the molecular sketch suggests that the S atom of the polymer is located on top of an Au atom position.

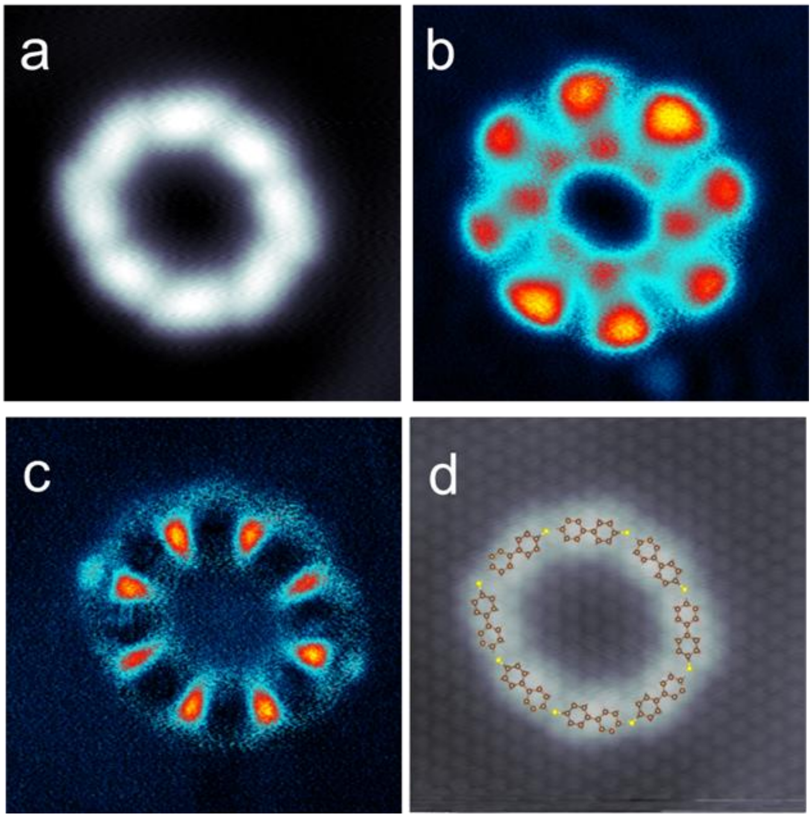

Figure SI6. Topographic and spectroscopic images of an octamer closed loop structure a. topographic image, and energy maps: **b**. 2.1V **c**.-1.5V , **d**. Superposition of topographic image (panel a) and atomic resolution of the Au(111) substrate at the same position after the removal of the ring. The structural model highlights the atomistic stacking configuration between S atoms, which are in the top position with respect to Au atoms. (Image size: 5nmx5nm)

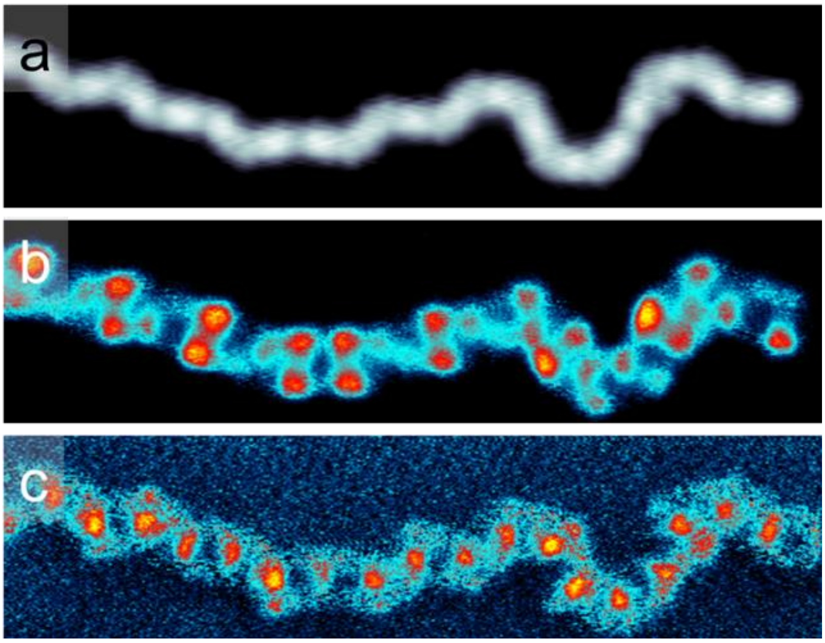

Figure SI7. Topographic and spectroscopic images of a chain structure a. topographic image (size: 15.2nmx3.8nm). Energy maps at **b.** 2.1V; **c.** -1.5V

5. Ph-S-Ph: electronic property

In Figure 2 of the main manuscript, we have shown that the electronic fingerprint of a Ph-S-Ph thioether structure is clearly visible at -1.5eV. The closed loop structure shown in figure 3 confirms this electronic character with the exception of the long and straight molecular segment formed by 4 consequent phenyl units as shown in SI8. This density of states is easily recognized far from the trapped Br atoms. It is, however, worth noting how this electronic structure can be observed only in the integer-thioether polymer structure. Indeed, when the molecular units are detached from the loop, the electronic fingerprint of the molecular sulfur in the C-S-C bond cannot be detected (arrows). A DFT-simulated structure of a 4-phenyl molecule with S terminations confirms the experimental data.

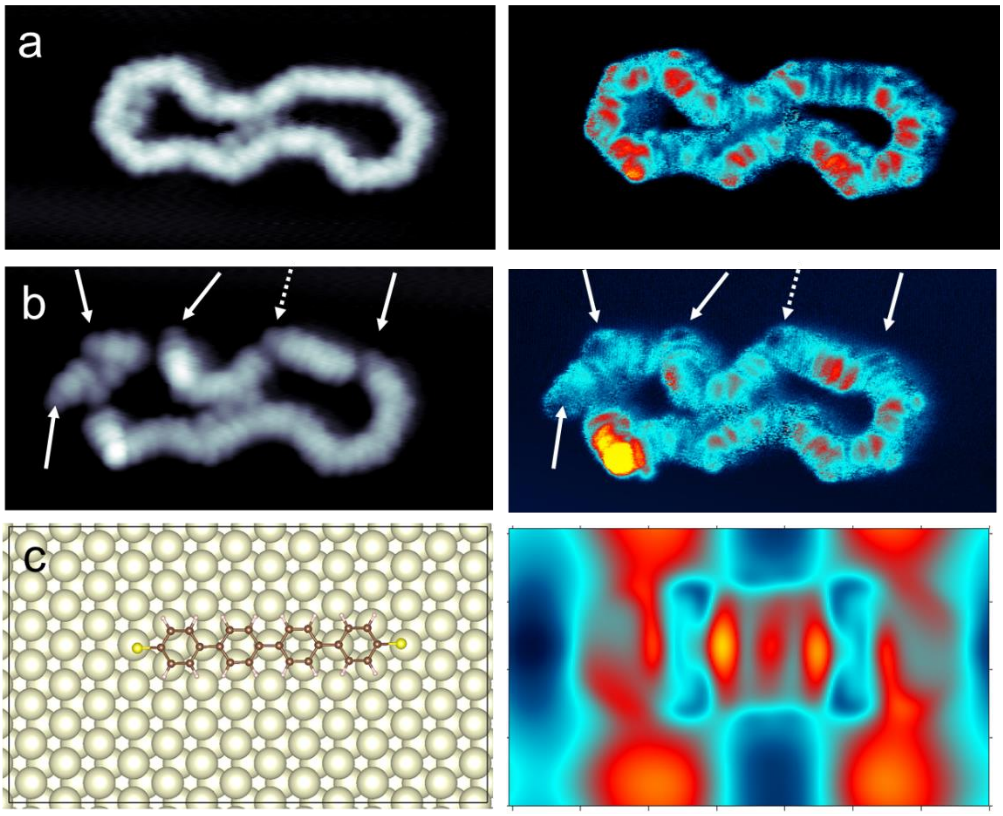

Figure SI8. The electronic fingerprint of a polymerized Ph-S-Ph at -1.5V. a. Topographic and energy map of the closed loop structure showing the absence of the S atom in the long linear segment. **b.** Closed loop structure dissociated into its molecular components. The arrows point to the terminal S positions. **c.** DFT simulation of a tetraphenyl molecule with S terminations and corresponding energy map.

5. References

[1] G. Kresse, J. Furthmüller, Efficiency of ab-initio total energy calculations for metals and semiconductors using a plane-wave basis set, *Comput. Mater. Sci.* 1996, 6, 15–50

[2] G. Kresse, D. Joubert, From ultrasoft pseudopotentials to the projector augmented-wave method, *Phys. Rev. B* 1999, 59, 1758–1775

[3] P. E. Blöchl, Projector augmented-wave method, *Phys. Rev. B* 1994, 50, 17953–17979

[4] J. P. Perdew, K. Burke, M. Ernzerhof, Generalized Gradient Approximation Made Simple, *Phys. Rev. Lett.* 1996, 77, 3865–3868

[5] A. Tkatchenko, M. Scheffler, Accurate Molecular Van Der Waals Interactions from Ground-State Electron Density and Free-Atom Reference Data, *Phys. Rev. Lett.* 2009, 102, 073005

[6] J. Tersoff, D. R. Hamann, Theory of the scanning tunneling microscope, *Phys. Rev. B* 1985, 31, 805–813

[7] M.-L. Bocquet, H. Lesnard, S. Monturet, N. Lorente, *In Computational Methods in Catalysis and Materials Science* (eds van Santen, R. A. & Sautet, P.) 199–219 (Wiley-VCH Verlag GmbH & Co.

[8] N. Lorente, R. Robles, STMpw, (Zenodo, 2019). https://doi.org/10.5281/ZENODO.3581159.

[9] K. Momma, F. Izumi, *VESTA 3* for three-dimensional visualization of crystal, volumetric and morphology data, *J. Appl. Crystallogr.* 2011, 44, 1272–1276

[10] A. Barragan, R.Robles, N.Lorente, L.Vitali, Power discontinuity and shift of the energy onset of a molecular de-bromination reaction induced by hot-electron tunneling,Nanoscale 2021, 13, 15215

[11] Abyazisani M., MacLeod J.M., Lipton-Duffin J., Cleaning up after the Party: Removing the Byproducts of On-Surface Ullmann Coupling, ACS nano 2019, 13, 9270
